# Size Changes in Honey Bee Larvae Oenocytes Induced by Exposure to Paraquat at Very Low Concentrations

**DOI:** 10.1371/journal.pone.0065693

**Published:** 2013-05-28

**Authors:** Marianne Cousin, Elaine Silva-Zacarin, André Kretzschmar, Mohamed El Maataoui, Jean-Luc Brunet, Luc P. Belzunces

**Affiliations:** 1 INRA, UR 406 Abeilles & Environnement, Laboratoire de Toxicologie Environnementale, Avignon, France; 2 Universidade Federal de São Carlos, Laboratory of Structural and Functional Biology, Sorocaba, São Paulo State, Brazil; 3 INRA, UR Biostatistiques et Processus Spatiaux, Avignon, France; 4 Université d’Avignon, Pôle Agrosciences, Avignon, France; French National Institute for Agricultural Research (INRA), France

## Abstract

The effects of the herbicide Paraquat were investigated in honey bee larvae with attention focused on oenocytes. Honey bee larvae were exposed to Paraquat at different concentrations in the food: 0, 0.001, 0.01, 0.1 and 1 µg/kg. In controls, between 24 h and 48 h, oenocytes grew from 630.1 to 1643.8 µm^2^ while nuclei changed in size from 124.9 to 245.6 µm^2^. At 24 h, Paraquat induced a slight decrease in the size of oenocytes and nuclei. N-acetylcysteine (NAC), an antioxidant substance, slightly lowered the effects of Paraquat. At 48 h, Paraquat elicited a strong concentration-dependent decrease in the size of oenocytes, even at the lowest concentration. NAC reversed the effect of Paraquat at a concentration of ≥0.01 µg/kg. This reversion suggested different modes of action of Paraquat, with an oxidant action prevalent at concentrations ≥0.01 µg/kg. This study is the first which reports an effect of a pesticide at the very low concentration of 1 ng/kg, a concentration below the detection limits of the most efficient analytic methods. It shows that chemicals, including pesticides, are likely to have a potential impact at such exposure levels. We also suggest that Paraquat could be used as a suitable tool for investigating the functions of oenocytes.

## Introduction

The honey bee *Apis mellifera* is a pollinator insect of agro-environmental, scientific and economic importance [1]. In order to gather food (pollen and nectar) and plant substances to make the antiseptic propolis, honey bees visit plants in a radius up to12 km around the hive, thus being exposed to a large variety of environmental stressors [2]. The honey bee is regarded as involved in 80% of global agricultural pollination and contributes to the plant productions by increasing the qualitative and quantitative yields of fruits and vegetables [3]. In 2009, the economic value of insect pollination for agriculture was estimated at close to 20 billion € per year in Europe and approximately 153 billion € worldwide, which represented 9.5% of the value of the world agricultural production used for human food in 2005 [4]. In the European Community, the direct value of honey produced is estimated at about € 140 million, and the total added value of the crop pollination service has recently been estimated at € 14.2 billion [5]. On a scientific level, the honey bee is considered as one of the best models for investigating cognitive functions such as vision, orientation, and memory [6], [7].

The constant decline of honey bee populations has been described and studied for the past 15 years [8]. Several factors have been identified to explain this decline, including pathogens and parasites, decrease of food resources and pesticides [9]. Environmental stressors, such as pesticides, may induce effects that impair not only the longevity but also the vitality of bees [5]. At very low doses, they may act at lethal and sublethal levels and elicit physiological, neural, metabolic and behavioral effects [10]. Sublethal effects can be very detrimental to the fate of the colony by altering the physiology and the functioning of individuals and by causing rapid depopulation.

Embryonic and post-embryonic developments are crucial phases during which environmental physiological disruptors may cause irreversible damages to the individuals. In the very early stages of honey bee larval development, trophocytes and oenocytes represent two main groups of cells that are easily observed in the larvae fat body [11]. Oenocytes exhibit a well-developed smooth endoplasmic reticulum and contribute to numerous physiological processes [12]. They are not only involved in the production of lipids and lipoproteins [13], but they also appear to be the source of precursors for cuticulin formation and play a role in the constitution of external cuticle in both larvae and adults [14]. In addition, they are involved in intermediary metabolism [15] and synthesize hydrocarbons to waterproof cuticle or to make beeswax [16], [17]. Furthermore, oenocytes secrete hormones, especially those involved in larval and adult development, such as the ecdysteroids that induce metamorphosis and trigger the remodeling of larval tissues [18]. They are also described as the major cells expressing NADPH-cytochrome P450 reductase (CPR) and are involved in detoxification of xenobiotics and in metabolic resistance to insecticides [19]. Some pesticides have been proved to modulate the number, size, structure and activity of oenocytes. Sublethal doses of the organophosphates Parathion and Demethon decrease the number and increase the volume of oenocytes in nymphs and adults of the aphid *Pemphigus bursarius* [20]. These changes are linked to the loosening of chromatin in the nuclei and the amitotic division of oenocytes. Dimilin, a chitin inhibitor, reduces the production of polysaccharides and consequently disturbs the formation of cuticle [21].

A few studies have focused on the toxicity of pesticides to brood and the effects have mainly been investigated on adult bees, with special attention to insecticides and fungicides. The recent release of a new method to analyze the toxicity to the honey bee brood enables more accurate researches on the effects of pesticides on embryonic and post-embryonic development [22], [23]. In the present study, the effects of a model substance, the herbicide Paraquat, on honey bee development have been examined by exposing the brood to very low concentrations of Paraquat in food. Although Paraquat was banned from the European Union in 2007 and in other countries, it is still used, at field rate of 0.3–2 kg/ha, on more than 100 crops in about 100 countries and particularly in developing countries [24]. Paraquat was shown to be non-toxic to slightly toxic to adult bees [25], [26]. Thus, the aim of this study was to see whether substances different from insecticides and generally considered as non-toxic to adult bees, such as herbicides, fungicides and insect growth regulators (IGR), can induce adverse effects on brood. The effects of Paraquat were also investigated in the presence of N-acetylcysteine (NAC), an aminothiol precursor of intracellular cysteine, which is a radical scavenger that exhibits antioxidant properties and attenuates the detrimental effects of Paraquat [27]. Paraquat has been chosen because it can elicit negative effects not related to its herbicide action in vertebrates and invertebrates. Paraquat, a quaternary ammonium herbicide of the bispyridinium family [28], is widely used in agriculture and can affect different non-target organisms such as fishes [29], Collembola [30], birds [31], Gastropoda [32] and mice [33] and is a non-specific inducer of apoptosis [34]. In human beings, Paraquat can trigger lethal lung fibrosis [35]. It is also strongly implicated in the development of Parkinson’s disease in farmers [36] and, acting as a pro-oxidant, can induce the formation of reactive oxygen species that elicit the destruction of dopaminergic neurons in the striatum [37].

## Materials and Methods

### Materials

Larvae of the honey bee *Apis mellifera* were randomly collected from three queen-right colonies, from the INRA apiary (Avignon, France), containing 45,000 to 50,000 bees and 6–7 brood frames. The honey bee colonies were carefully monitored to check their health status. The day before the experiment, empty combs were inserted in hives. Twenty four hours after, cells containing eggs were identified by means of a transparent grids and the combs were left in the hives for 3 days. In the early morning of day four, the combs were transported into the laboratory and newly hatched larvae, within previously identified cells, were exposed to Paraquat for two days in laboratory. The effects of Paraquat on oenocytes were observed after 24 and 48 h of exposure, which means that exposure to Paraquat through the food was perfectly controlled during the first two days of observation. Two brood combs per hive were collected to facilitate the gathering of 200 larvae for each experimental group, and were placed in rearing boxes with controlled temperature (33±1°C) and relative humidity (90%). Paraquat (98% pure; 1,1′-Diméthyl-4,4′-bipyridinium; CAS number: 1910-42-5) was purchased from Cluzeau-Info Labo, France. The concentrations of the stock solutions of Paraquat were checked by high-performance liquid chromatography with a method optimized for Paraquat solutions that enables reaching a limit of quantification of 8 ng/L and a limit of detection of 2.4 ng/L in simple matrices [38], [39]. The difference between nominal and measured concentrations did not exceed 4%. Yeast extract (Y1000) and N-acetylcysteine were obtained from Sigma-Aldrich (France).

### Experimental procedures

The marked larvae were randomly divided into five groups of 200 larvae (3 colonies, 65–67 larvae per colony and per Paraquat concentration), each of which were exposed to a specific concentration of Paraquat, namely: 0 (Control), 0.001, 0.01, 0.1 and 1 µg/kg of food, during larval development, in the presence (NAC) or in the absence (NoNAC) of N-acetyl-cysteine (NAC) at a concentration of 50 µg/kg. Thus, for each treatment condition (Paraquat at 5 concentrations, plus or minus NAC), 32–34 bees were individually analyzed per colony. The larvae from each colony were clearly identified for statistical analysis. The larvae were fed three times, at 0, 24 and 48 h, with a basic diet containing 50% (v/v) of royal jelly and 50% (v/v) of an aqueous (distilled water) solution containing yeast extracts (1%, w/v), D-Glucose (6%, w/v) and D-fructose (6%, w/v) [40]. Paraquat was added directly, from stock solutions in water, into the distilled water used for food preparation. For each marked larva, food was delivered by means of a micropipette with a volume of 5 µL at 0 h, 8 µL at 24 h and 10 µL at 48 h of larval development.

### Histochemistry

For each experimental group, larvae were randomly sampled at 24 and 48 h (they were fed once or twice respectively) and separately prepared for light microscopy. For morphological analysis, the larvae were fixed in 4% (v/v) formaldehyde in 0.1 M sodium phosphate buffer pH 7.2 for 48 h at 4°C. Larvae were then rinsed in a phosphate buffer, sequentially dehydrated in ethanol [from 70% to 100% (v/v)] and embedded in resin (Kit Technovit 7100; Heraeus-Kulzer GmbH, Wehrheim, Germany) according to the instructions of the manufacturer. Tissue sections (3 µm thick) were obtained using a retraction microtome (Reichert-Young Supercut 2065, Vienna, Austria), collected on microscope slides, dried and stored in dust-proof containers until staining. During the next stages, the slides were hydrated in distilled water before staining in order to visualize polysaccharides and proteins using periodic acid-Schiff's reagent (PAS) and naphtol blue black procedures, respectively [41]. After staining, the slides were dried and cover slipped in Surgipath Micromount medium (Surgipath, Richmond, Canada). Sections were photographed using a light microscope VWR (TR500 High End Tri.) at 100X magnification.

### Oenocyte analysis

Images were processed with a Motic Images Plus camera 2300. For each group of treatment, all the oenocytes of each section from the different larvae were analyzed using ImageJ software 1.43u (US NIH) to measure the cell (Ac) and nucleus (An) areas. The mean number of oenocytes analyzed per larvae was 81±30. The nucleo-cytoplasmic ratio NCR was estimated by the following formula:

NCR  =  An/(Ac – An)

For the global statistical analysis, data were processed by a generalized linear model (GLM) with a log link function for analyzing the effect of the different experimental factors (i.e., larvae stage, Paraquat concentration and NAC). In order to assess the effects of the factor levels, statistical differences between treatment groups were analyzed with a multi-T-test, after normality had been checked to fit the distribution of observed measurements at each factor level. For non-independent tests, a Bonferroni correction was applied. The level of significance was determined when the *p* value was greater than 0.001. The concentration-effect relationship of Paraquat after 48 h of exposure was explored by log-log linear regression analysis.

## Results

### NCR and cell size in control oenocytes

**Figure 1:**
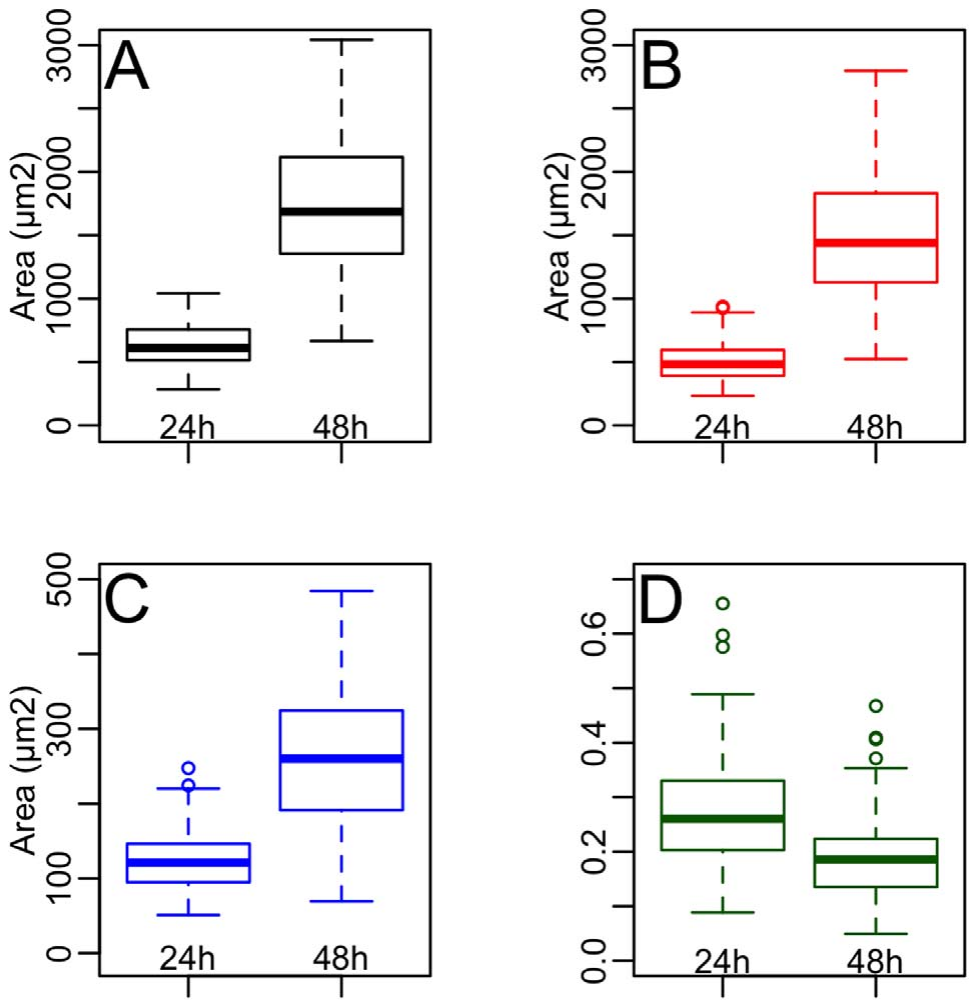
Evolution of oenocytes between 24 and 48 h. Changes of oenocyte biometrical parameters were noticed at 24 h (n = 138) and 48 h (n = 169) and were expressed as areas (µm^2^). At the given time, larvae were picked out, immediately fixed in formaldehyde and processed for histochemical analysis. Oenocytes were detected and total cell and nuclei areas were measured on each oenocytes. Three compartments were analyzed : whole cell (A), cytoplasm (B, by subtracting nucleus area from total cell area) and nucleus (C), and the nucleo-cytoplasmic ratio NCR (D) was calculated. Surface values distribution at 24 and 48 h were compared with pair T-tests which assess significant differences for all parameters studied (p<1.10^−5^).

At concentrations used in this study, Paraquat did not induce significant mortality in honey bee larvae (T test: p value  = 0.471). Significant growth of oenocytes was observed between 24 and 48 h with respective sizes of 630.12±175.96 and 1643.77±479.02 µm^2^ (T-test: p value  = 2.2.10^−16^) (Fig. 1). In the same period, the size of the nuclei increased significantly from 124.89±38.95 to 245.55±89.74 µm^2^ (T-test: p value  = 2.2.10^−16^). As a consequence, the variation of NCR between 24 and 48 h decreased significantly from 0.269±0.106 to 0.187 ±0.075 (T-test: p value  = 5.982.10^−13^).

### Effects of Paraquat on oenocytes

The global analysis with GLM showed significant effects of the experimental factors on the different oenocyte compartments. In the case of entire cells, larvae stage and Paraquat concentration had a *p* value <2.10^−16^ while the *p* value for NAC was 0.0287. Similar results were obtained for the cytoplasm compartment whereas, for nuclei, there was no significant effect of NAC. Additionally, no effect of larvae stage was observed for NCR whereas the other factors were highly significant (*p* value <2.10^−16^ and 1.39.10^−8^ for Paraquat concentration and NAC, respectively).

**Figure 2:**
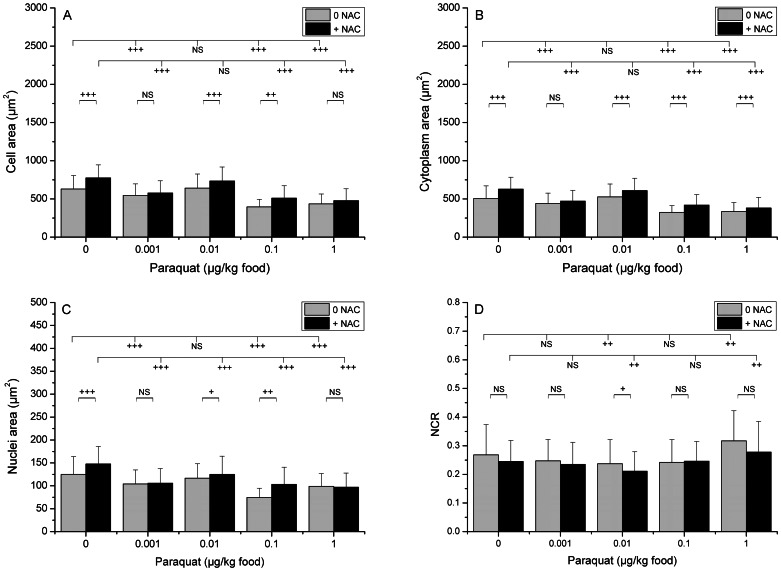
Effects of Paraquat at 24 h on the size of three oenocyte compartments. Larvae were exposed at different concentrations of Paraquat (0 (control), 0.001, 0.01, 0.1 and 1 µg/kg of food) in the absence (NoNAC) or in the presence of NAC. They were then picked out, immediately fixed in formaldehyde and processed for histochemical analysis. Statistically significant effects were observed after 24 h of exposure to Paraquat. Three compartments were analyzed: whole cell (A), cytoplasm (B, by subtracting nucleus area from total cell area) and nucleus (C), and the nucleo-cytoplasmic ratio (NCR) was calculated (D). (+), *p*<0.01; (++), *p*<0.001; (+++), *p*<0.0001 ; NS, not significant (*p*>0.05).

**Figure 3:**
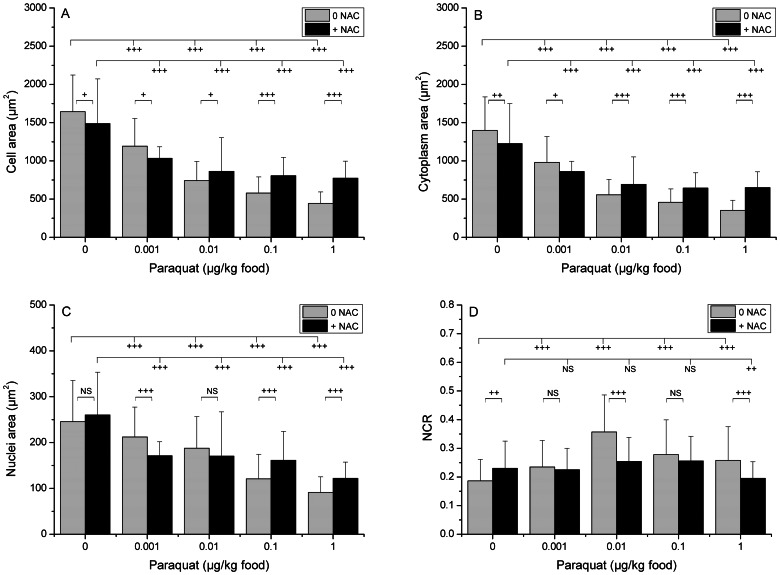
Effects of Paraquat at 48 h on the size of three oenocyte compartments. Larvae were exposed at different concentrations of Paraquat (0 (control), 0.001, 0.01, 0.1 and 1 µg/kg of food) in the absence (NoNAC) or in the presence of NAC. Then, they were picked out, immediately fixed in formaldehyde and processed for histochemical analysis. Biometrical effects were observed after 48 h of exposure to Paraquat. Three compartments were analyzed, whole cell (A), cytoplasm (B, by subtracting nucleus area from total cell area) and nucleus (C), and the nucleo-cytoplasmic ratio (NCR) was calculated (D). (+), *p*<0.01; (++), *p*<0.001; (+++), *p*<0.0001 ; NS, not significant (*p*>0.05).

**Figure 4:**
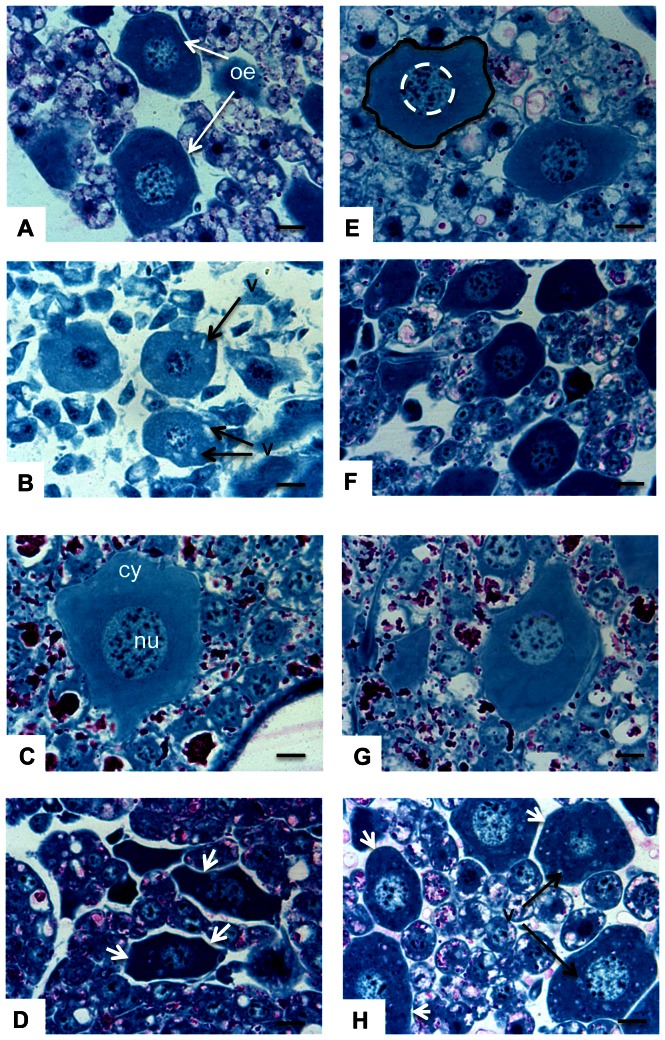
Effects of Paraquat on oenocyte morphology. Larvae were subjected to exposure to Paraquat at different concentrations in the food in the absence (A, B, C, D) or in the presence (E, F, G, H) of NAC for 24 and 48 h. Larvae were then fixed in formaldehyde 4% (v/v), dehydrated in ethanol and embedded in methacrylate resin. Sections (3 µm) were stained with the PAS-NBB procedure and observed under light microscopy. (A and E), controls at 24 h. (A), identification of oenocytes. (E), method of area measurement: black line, total area, and white dotted line, nuclei area. (B and F), oenocytes at 24 h in larvae exposed to Paraquat at 1 µg/kg. Notice the smaller nuclei and cytoplasm areas by comparison with controls (A and E) and also small vacuoles in the cytoplasm (black arrow). (C and G), controls at 48 h. Observe the increase in cell and nuclei areas between 24 h (A and E) and 48 h. (D and H), oenocytes at 48 h in larvae exposed to Paraquat at 1 µg/kg. Note the smaller nuclei and cytoplasm areas by comparison with controls (C and G) and also small vacuoles in the cytoplasm (black arrow). The cytoplasm of oenocytes from larvae exposed to Paraquat was more contracted in the absence of NAC (D, white arrow heads) than in the presence of NAC (H, white arrow heads). cy  =  cytoplasm, n  =  nuclei, oe  =  oenocyte and v  =  vacuole. Scale bar  = 10 µm; magnification: 600.

The effects of Paraquat on biometrical parameters of oenocytes were studied in the absence (noNAC) and in the presence (NAC) of N-Acetylcysteine, an antioxidant substance that prevents the cells from the oxidative effects of Paraquat (Fig. 2, 3 and 4).

At 24 hours, in the absence of NAC, Paraquat induced a slight decrease in the size of oenocytes (except at the concentration of 0.01 µg/kg) and nuclei (Fig. 2). Consequently, a decrease in the cytoplasm area was observed only at the two highest concentrations (0.1 and 1 µg/kg) (Fig. 2, and 4A, 4B, for 1 µg/kg), which therefore resulted in an increase of NCR at the highest concentration. NAC only slightly modified the effects induced by Paraquat at 24 h and, like in the absence of NAC, a decrease of cell and nuclei sizes was observed in the presence of NAC (Fig. 2). However, at 24 h, NAC slightly attenuated the reduction of size of cells and nuclei induced by Paraquat (Fig. 2 and 4A, B, E, F). Thus, as a result, NCR remained nearly stable.

At 48 h, in the absence of NAC, Paraquat had a major significant concentration-dependent effect upon oenocytes by decreasing the size of the cell, cytoplasm and nucleus (Fig. 3). Concentration-effect relationship was explored with log-log linear regression analysis (adjusted R-square 0.9785) and enabled to determine an effective concentration 50% of 16.00±2.24 ng/kg. It is noteworthy that a significant decrease was already observed at the lowest concentration (0.001 µg/kg). The size of entire cells was reduced by 50% at the concentration of 0.01 µg/kg, compared to the control. The intensity of the size decrease at the two highest concentrations was less marked because the decrease of the cell volume is limited by the nucleus (Fig. 4D). The concentration-dependent decrease of nuclei area presented a more regular pattern and, consequently, NCR increased with the concentration. The decrease elicited at the highest concentration was important enough to almost abolish the cell growth between 24 and 48 h: 630.12 µm^2^ and 1643.77 µm^2^ for controls at 24 and 48 h respectively and, 434.48 µm^2^ and 443.55 µm^2^ for 1 µg/kg food at 24 and 48 h, respectively. At 48 h, the average decrease in size due to the highest concentration compared with control (73.0% and 62.9% for total cell and nuclei areas, respectively) was more than twice the decrease at 24 h (31.05% and 20.83% for total cell and nuclei areas, respectively) (Fig. 2 and 4C, D). When the effect of Paraquat was observed in the presence of NAC at 48 h, NAC in turn had a significant effect (Fig. 2 and. 4C, D, G, H). NAC attenuated the Paraquat-induced decrease of cell and cytoplasm areas at the three highest concentrations and the nuclei area at the two highest concentrations. For the control and for the lowest Paraquat concentration (0.001 µg/kg), NAC induced a slight reduction in the size of the cell but not of the nuclei. As a consequence, cytoplasm and total cell areas presented identical concentration-response patterns. It is notable that oenocytes from exposed larvae, but not from controls, exhibited vacuoles in the cytoplasm (Fig. 4).

## Discussion

In the honey bee, oenocytes have been described early at different developmental stages [42]. The size of these cells increases gradually during the larval development, as in other species such as *Drosophila* [43]. In the present study, we show that Paraquat induces a concentration-dependent decrease of total cell and nuclei areas, to such a degree that the growth between 24 and 48 h (usually about tripling in size) does not take place. The effect of Paraquat is also observed at the lowest concentration (0.001 µg Paraquat/kg food), which demonstrates the high sensitivity of oenocytes. This concentration is indeed very low and much lower than the limit of detection, in complex matrices, of very efficient analytical methods involving mass spectrometry [44]. In other words, it will be impossible to detect Paraquat in larvae food even at higher concentrations close to 100 ng/kg, for which a significant effect is observed. Thus, this is the first time that biological effects of pesticides can be observed at such very low exposure levels.

The effects of Paraquat demonstrated here raise the question of the impacts of quaternary ammonium herbicides, and especially pyridinium active substances, on the development of the honey bee. The herbicide Diquat also triggers an oxidative stress [45] and can accumulate in the neuromelanin of frogs and mice [46]. MPP+ (1-methyl-4-phenyl pyridinium), a neurotoxic metabolite of the drug MPTP (1-methyl-4-phenyl-1,2,3,6-tetrahydropyridine), has been registered as the herbicide Cyperquat [47]. Like Paraquat and rotenone, it induces Parkinson’s disease by destroying the dopaminergic neurons in the *substantia nigra* but acts through a mechanism close to that of rotenone and different from that of Paraquat [48]. The honey bee brain is devoid of structure like *substantia nigra* [49], and a different mechanism of action could be expected for Paraquat in the honey bee larvae. However, recent results suggest that Paraquat may exhibit common action pathways in the honey bee and mammals. Paraquat induces the condensation of cytoplasm and irregular cell shapes not only in bees, but also in mouse hippocampus cells [50]. These changes are associated with a decay of learning and memory performances and the induction of an oxidative stress in mouse. Thus, it would be worth investigating the effects of Paraquat on larvae cells, and especially neural cell precursors, and their impacts on neural functions in adult bees.

The accentuated presence of vacuoles in the cytoplasm of oenocytes from exposed larvae, but not from the controls, could be interpreted as a primary sign of cell death in insect tissue [51] that could impair hormone synthesis. In addition, oenocytes are involved in the synthesis of ecdysteroid hormones that regulate different genes [*AmEcR-A, AmE74*, *AmE75*, *AMBR-C*, *AmHR-38*, *Mblk-1 (AmE93)* and *AmUsp (Ultraspiracle)*] preferentially and differentially expressed in the mushroom bodies of the queen and the worker brain, particularly in the Kenyon interneurons [52], [53]. Since Kenyon cells are regulated by ecdysteroids and involved in cognitive functions and brain plasticity [54], [55], it would be interesting to further explore the cognitive effects of Paraquat in adult bees exposed at larval stages. However, the influence of Paraquat should not be restricted to neural functions and the fertility of the queen should also be taken into consideration because *AmEcR-A* and *AmE74* genes are strongly expressed in the ovary of the queen [52], [56].

NAC is known as an antioxidant substance and a free radical scavenger [57]. In the honey bee larvae, the protective action of NAC against the Paraquat-induced effects in oenocytes is more pronounced after 48 h of exposure than after 24 h. At 48 h, NAC moderates the Paraquat-induced reduction of the size of cells and cytoplasm at Paraquat concentrations ≥0.01 µg/kg, and of nuclei at Paraquat concentrations ≥0.1 µg/kg. In this study, NAC shows a protective effect at a concentration of 50 µg/kg, which is 100 to 1000 times lower than the concentrations used in studies on Paraquat in vertebrates [58]. Thus, at this concentration, NAC also seems to present antioxidant properties to counteract the oxidative damages induced by Paraquat in honey bee larvae. Yet, the effects of Paraquat are moderated but not completely abolished by NAC, whatever the Paraquat concentration. This reveals that Paraquat has a strong effect on larvae cells, even at very low concentrations. NAC displays a differential action at low and high Paraquat concentrations. From 0.01 to 1 µg/kg Paraquat, NAC reduces the effect of Paraquat whereas in controls and at 0.001 µg/kg, it decreases the cell and cytoplasm areas. This suggests different modes of action of Paraquat, depending on the concentration, with an oxidant action of Paraquat prevalent at concentrations above 0.01 µg/kg.

In this study, we have demonstrated the high sensitivity of oenocytes to Paraquat at very low concentrations. However, considering that the Paraquat-induced effects in bees are also observed in mammalian brain cells [50], it is legitimate to think that they could also occur in other cell types and at different developmental stages. Hence, changes in oenocyte morphology could be regarded as a high sensitivity biomarker of exposure to pesticides and as a biological model to study the effects of these latter. Very few cells have been described as markers of intoxication in honey bees, especially, midgut epithelial cells and Malpighian tubule cells [59]. Oenocytes are large and independent cells embedded in fat body and readily accessible [16] and thus represent a good study model [42]. The sensitivity of oenocytes, and the very low concentrations at which Paraquat induces effects, indicate that these cells could respond not only to direct exposure but also to pollen and wax contamination [60], [61].

## Conclusion

In this study, we have established that Paraquat, a model pesticide, can disrupt the larval development of oenocytes at very low concentrations. The most important point is that concentrations around ng/kg are able to induce damages in living organisms and especially in honey bees. Since a model molecule has proved to be efficient at this contamination level, it seems legitimate to think that the same phenomenon is likely to take place with other pesticides. Regarding the sensitivity of oenocytes to low concentrations of Paraquat, we advance that the morphology of oenocytes could be used in developing biomarkers of exposure to pesticides. The development of this biomarker will require further in-depth studies with other chemicals, at other developmental stages and in relatively large scales involving spatial and temporal variations. The impairment of oenocyte development at very low exposure levels makes Paraquat a valuable tool for the investigation of the functions of oenocytes.
